# An unusual case of redo tricuspid valve replacement and repair of a previously unidentified anomalous pulmonary venous return in a patient with congenitally corrected transposition of the great arteries

**DOI:** 10.1002/ccr3.2902

**Published:** 2020-04-27

**Authors:** Amir H. Sadeghi, Pieter Van de Woestijne, Yannick J.H.J. Taverne, Arie P. J. Van Dijk, Ad J. J. C. Bogers

**Affiliations:** ^1^ Department of Cardiothoracic Surgery Academic Center for Congenital Heart Disease Erasmus University Medical Center Rotterdam The Netherlands; ^2^ Department of Cardiology Academic Center for Congenital Heart Disease Radboud University Medical Center Nijmegen The Netherlands

**Keywords:** congenitally corrected transposition of the great arteries, congenital cardiac malformation, partial anomalous pulmonary venous return, redo cardiac surgery

## Abstract

Associated cardiovascular malformations in congenitally corrected transposition of the great arteries (CCTGA) should not be missed when a patient requires surgical correction. We present a case of an adult CCTGA patient who required redo surgery for recurrent tricuspid (left atrioventricular) valve regurgitation and previously unidentified partial anomalous pulmonary venous return.

## Graphical abstract

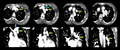

## 1 INTRODUCTION

Congenitally corrected transposition of the great arteries (CCTGA) is a rare congenital heart defect characterized by both atrioventricular (AV) and ventriculo‐arterial (VA) discordance. The left atrium is connected (via a tricuspid valve) to the morphologic right ventricle, which is connected to the aorta.^1^, ^2^ CCTGA can be associated with other intracardiac malformations, including atrial septal defects (ASDs), ventricular septal defects (VSDs), Ebstein's anomaly of the left AV (tricuspid) valve, (sub)pulmonary stenosis, and it may be associated with other rare abnormalities such as anomalous pulmonary venous return (APVR) as well.^2^, ^3^ A small percentage (~10%) of patients with CCTGA do not have any associated cardiac malformations and therefore remain asymptomatic until adulthood. However, these patients often present during adulthood with symptoms secondary to systemic (morphologic) right ventricular dysfunction/failure, tricuspid (left AV) valve regurgitation, and conduction abnormalities.^1^, ^2^, ^4^ The latter is often due to abnormalities in the position of the AV‐node, which results in an increased chance of complete heart block.^5^

When CCTGA patients present, they usually require surgical intervention, especially when there are signs of systemic ventricular deterioration, such as ventricular dilatation, pulmonary congestion, and arrhythmias.^2^ However, associated intracardiac lesions may cause earlier symptoms and clinical manifestation, which may require (surgical) intervention at an earlier stage. Most commonly, procedures such as tricuspid (left AV) valve repair/replacement are performed, and physiologic repair of associated cardiac defects, such as ASD, VSD, and Partial‐APVR (PAPVR), should be considered when these defects are present.^2^, ^6^

Here, we present a unique surgical case of incidental detection of a rare left‐sided PAPVR in a 33‐year‐old male patient with situs solitus, CCTGA, who had previously undergone surgical ASD closure and tricuspid (left AV) valve repair because of tricuspid regurgitation. The aim of this case report was to present a rare case of a PAPVR in a patient with CCTGA and to emphasize the importance of complete preoperative workup and imaging in these patients.

## 2 CASE REPORT

**Figure 1:**
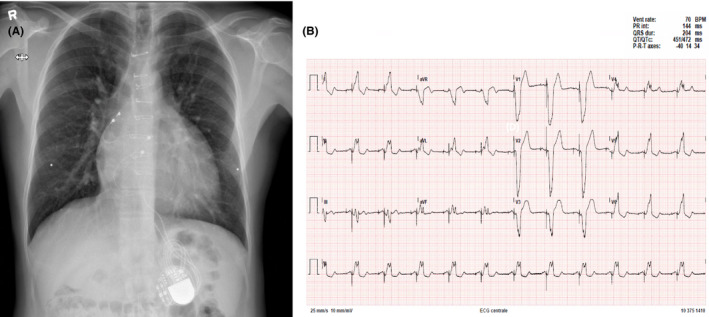
A, Conventional chest X‐ray demonstrating sternal metal suture wires, an epicardial pacemaker system with an abdominal generator, and without pathophysiological abnormalities. B, Preoperative ECG shows atrioventricular‐paced rhythm with a ventricular contraction rate of 70 beats per minute (BPM)

**Figure 2:**
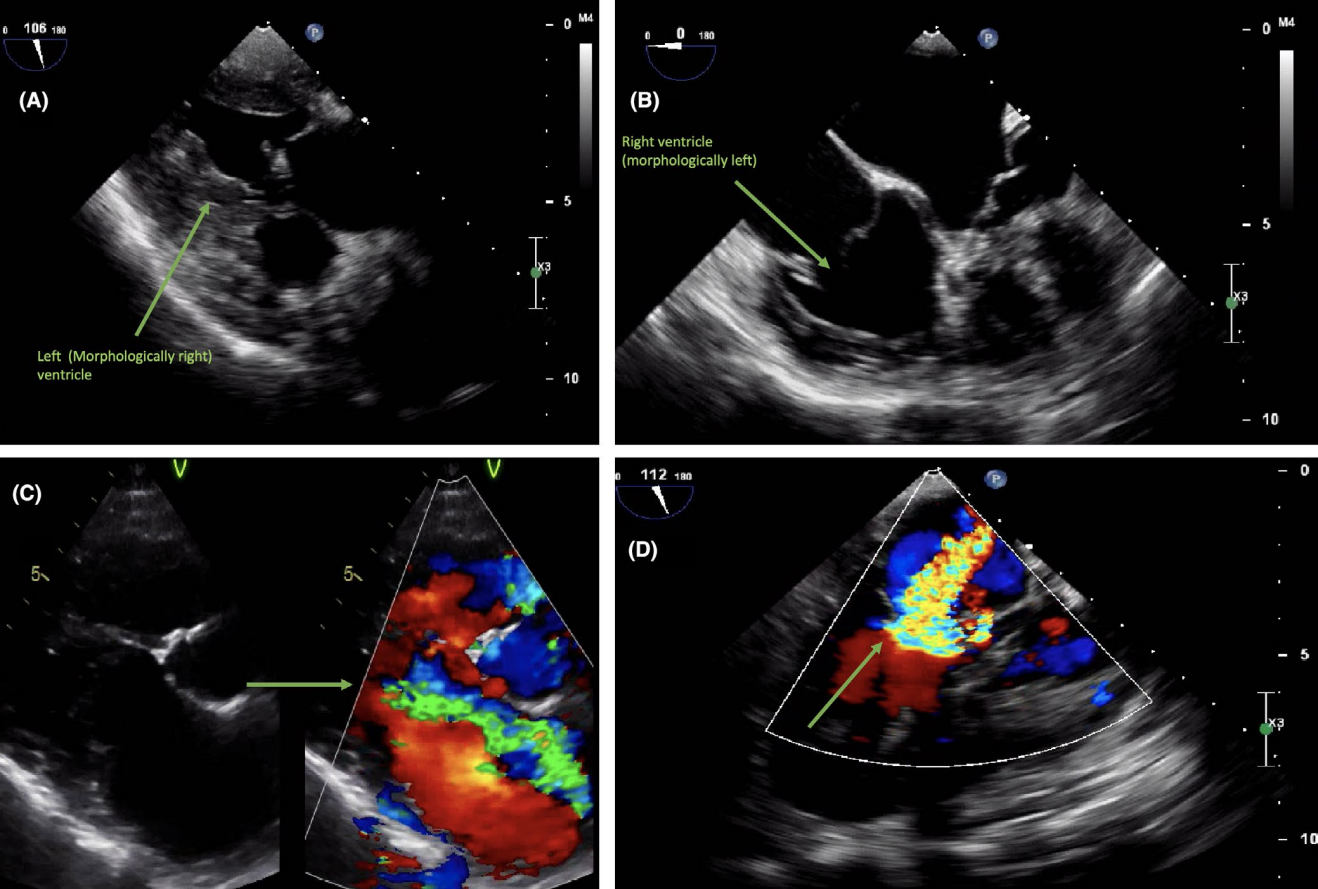
Transesophageal and transthoracic echocardiography views of A, left (morphologically right) ventricular hypertrophy, B, right (morphologically left) ventricular dilatation, and C and D, severe tricuspid (left atrioventricular) valve regurgitation by color‐doppler flow views

**Figure 3:**
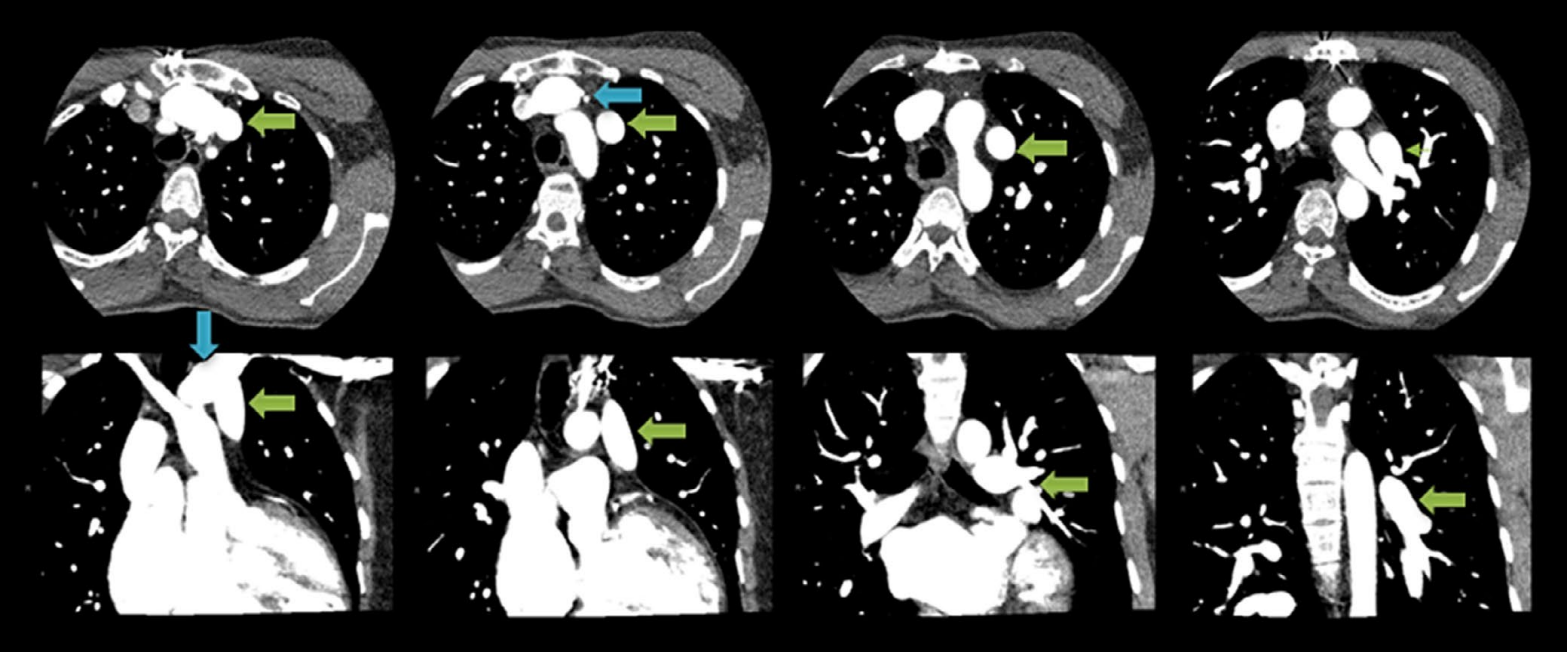
Preoperative CT‐angiogram scan in coronal (bottom panel) and transversal plan (top panel), demonstrated left pulmonary vein (PAPVR) (green arrows) draining into left brachiocephalic vein (blue arrow)

In 2007, a 22‐year‐old male patient with CCTGA underwent an ASD closure, an epicardial dual chamber pacemaker system placement, and a tricuspid (left AV) valve repair for severe tricuspid valve regurgitation, atrioventricular block, and an ASD associated with his CCTGA. In 2018, this patient (now 33 years old) was referred to our institution for surgical treatment of recurrent tricuspid valve ((left AV)) regurgitation. The patient had progressive dyspnea on exertion and persistent symptoms of fatigue due to severe systemic valve regurgitation and ventricular deterioration. Preoperative electrocardiogram showed atrioventricular‐paced rhythm, and conventional chest X‐ray showed no abnormalities (Figure 1). Transthoracic and transesophageal echocardiography revealed dilatation of the right heart chambers (morphologic left ventricle), severe tricuspid (left AV) valve regurgitation, and left (morphologic right) ventricular hypertrophy with a moderately reduced ejection fraction (Figure 2). To clarify the exact anatomy and to prepare for resternotomy, a computed tomography angiography of the thorax was performed. The CT scan revealed a coexisting and previously unidentified partial anomalous pulmonary venous return (PAPVR) in which the left pulmonary vein was draining completely into the right atrium through superior vena cava and the left brachiocephalic vein (Figure 3). The patient was accepted for surgical treatment, and informed consent was obtained. The most important indication for surgery was severe tricuspid ((left AV)) valve regurgitation. The indication for PAPVR repair was based on dilatation of the right heart chamber (morphologic left ventricle) as a result of volume overload, most likely due to PAPVR and previously existing ASD.

**Figure 4:**
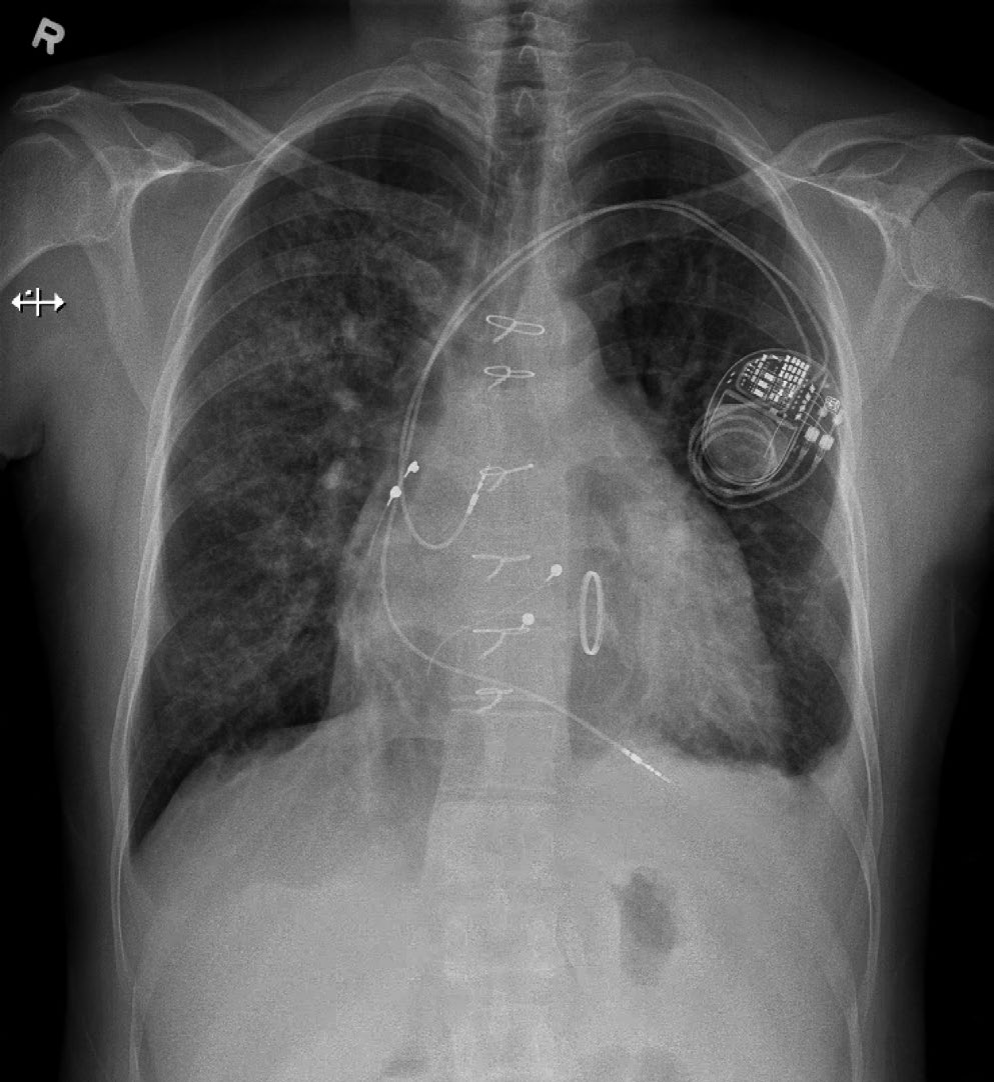
Conventional chest X‐ray image of the patient after tricuspid (left atrioventricular) valve replacement with a mechanical prosthesis and after transvenous pacemaker lead (dual chamber) placement. In addition, there are some signs of minimal left‐sided pleural effusion and bilateral interstitial lung edema

The patient underwent an elective redo operation through median sternotomy. During redo sternotomy, the epicardial pacemaker leads were damaged and removed. Before initiating cardiopulmonary bypass, the connecting left‐sided PAPVR was identified. Subsequently, cardiopulmonary bypass was initiated and cardioplegic cardiac arrest applied. Firstly, the patient's tricuspid (left AV) valve, which was macroscopically severely degenerated, was replaced with a 27mm St. Jude mechanical prosthesis. Then, the PAPVR was reimplanted from the brachiocephalic vein on the left atrial appendage, through a box‐like pericardial window posterior to the left phrenic nerve. Finally, temporary epicardial pacing wires were placed as a bridge to permanent transvenous pacemaker leads. The postoperative course was uneventful. Five days after surgery, permanent transvenous pacing leads were placed (Figure 4), and after eight days, the patient was transferred to the referring hospital. One day later, he was discharged home.

## 3 DISCUSSION

In this report, we present a unique case of a 33‐year‐old male patient with CCTGA that was associated with PAPVR that was previously (11 years ago) unidentified during tricuspid (left AV) valve repair and ASD closure.

PAPVR is a rare congenital malformation and occurs when pulmonary veins drain into the right atrium (or one of its venous tributaries, such as vena cava) instead of the left atrium. It has been stated that anomalous pulmonary veins are mostly right‐sided, meaning that most of these anomalous veins arise from the right pulmonary lobes.^7^ Examples of these right‐sided abnormalities include anomalous veins in scimitar syndrome (right‐sided PAPVR connecting directly to the inferior vena cava/right atrium) and superior caval ASD. In contrast, left‐sided PAPVR, as present in this case, is a rare condition and has been reported in 3%‐8% of the cases.^8^ Various types of isolated PAPVR have been described in the literature, among which the PAPVR with coexisting superior caval ASD (sinus venosus malformation) is the most common variant.^9^

The diagnosis of PAPVR is quite rare, since PAPVR is a malformation that is most often asymptomatic and does not always require repair.^10^ Also, when symptomatic, the patient's clinical manifestation and symptoms (such as dyspnea, fatigue, palpitations, syncope, and arrhythmias) are not always specific to PAPVR.^11^ Consequently, (noninvasive) imaging modalities are essential in order to diagnose or to confirm patients with PAPVR. In patients undergoing (congenital) cardiac surgery, transthoracic/transesophageal echocardiography is often obtained to evaluate cardiac function, valvular abnormalities and to confirm other associated intracardiac malformities. In a study by Wong and colleagues, it was shown that the diagnosis of PAPVR (in a pediatric population) was missed in 33% of the cases by using only transthoracic echocardiography.^12^ Even though transesophageal echocardiography is a more sensitive method to confirm the diagnosis of PAPVR, the results are very user‐dependent and insufficient information might be obtained on the exact anatomical details of PAPVR.^13^ However, in order to reliably confirm the presence and characteristics of anomalous pulmonary veins and to quantify associated left‐to‐right shunt, other imaging modalities such as multidetector computed tomography (MDCT) and magnetic resonance imaging (MRI) are required.^11^, ^14^, ^15^, ^16^ In the present article, a left‐sided PAPVR in a CCTGA patient was not identified during the index operation 11 years ago. This could be explained by the fact that no preoperative CT or MR scan was performed at that time. Without the existence of right‐sided valvular dysfunction or pulmonary hypertension, signs of right ventricular deterioration (such as dilatation or dysfunction) should often raise the suspicion of an important left‐to‐right shunt. In our patient, however, there was a coexisting ASD which was, at that time, possibly expected to be the predominant factor for right heart dilatation.

In patients with PAPVR, the general principle of surgical correction is based on replanting the anomalous pulmonary vein onto the left atrium either indirectly (to the left atrium through intracardiac baffles or GORE‐TEX™ grafts^10^, ^12^, ^17^ or directly by anastomosing the (left‐sided) PAPVR onto the left atrium or left atrial appendage, as occurred in this case. PAPVR repair is a relatively low‐risk operation that can be accomplished with minimal morbidity or mortality.^18^ When isolated PAPVR is present in patients, a relatively benign course is expected and surgery is not always required if patients lack signs of right‐sided heart dysfunction. However, in a single‐center study of adult patients (n = 43) with isolated PAPVR who required surgical repair, it was shown that there were improvements in echocardiographic measurements of pulmonary artery pressures and right ventricular function.^19^

In patients with CCTGA, a thorough and complete knowledge of the anatomy seems very important prior to open heart surgery. Although CCTGA is often associated with more common congenital malformities (eg, VSD, ASD), cases of TAPVR and their surgical corrections have also been described in the literature.^3^, ^6^ To the best of our knowledge, however, we report a very rare and unique case of redo surgery of a left‐sided PAPVR after tricuspid (left AV) valve repair and ASD closure in a patient with CCTGA.

Since the combination of VA and AV discordance maintains a “normal” physiological circulation, CCTGA patients without other important heart malformations could remain asymptomatic until adulthood. The exact timing of clinical presentation of adult CCTGA patients depends on the associated intra‐ and extracardiac malformations. Most commonly, patients are referred for open heart surgery due to symptoms secondary to morphologic right (systemic) ventricular dysfunction and tricuspid (left AV) valve regurgitation. Primary tricuspid (left AV) valve regurgitation could mostly be attributed to the often associated Ebstein's anomaly of the tricuspid valve. However, tricuspid (left AV) valve regurgitation could also be secondary to dysfunction of morphologic right (systemic) ventricle. In this case, the patients’ first clinical presentation was at the age of 22 because of symptoms secondary to tricuspid (left AV) valve regurgitation. During the index operation, an associated ASD closure was also performed and an epicardial pacemaker system was placed. 11 years later, at the age of 33, the patient was referred again due to recurrence of severe tricuspid (left AV) valve regurgitation and tricuspid valve replacement was carried out. Moreover, a previously unidentified left‐sided PAPVR was also corrected.

## 4 CONCLUSION

CCTGA is often associated with other cardiac and extra‐cardiac congenital malformations. CCTGA patients often present during adulthood due to symptoms that are associated with left‐sided ventricular dysfunction or left atrioventricular valve (tricuspid valve) regurgitation. When an adult patient presents with symptoms or echocardiographic signs of right (morphologic left) ventricular deterioration (eg, right ventricular dilatation), this should raise the suspicion of an important left‐to‐right shunt. CCTGA is more commonly associated with atrial and ventricular septal defects; however, the coexistence of an anomalous pulmonary venous return should not be missed when right‐sided ventricular dysfunction or dilatation is present. It is recommended to use echocardiography and additional imaging techniques (such as CT/MRI) to evaluate cardiac function and to identify associated malformations in order to prepare for surgical correction. This case stresses the importance of a thorough and complete preoperative workup and imaging in all patients who are accepted for surgical treatment, especially patients with CCTGA.

## CONFLICT OF INTEREST

None declared.

## AUTHOR CONTRIBUTIONS

All authors: made substantial contribution and have approved the final version of this manuscript. All authors: made substantial contribution to the treatment and operation of the patient described in this case. PW and AS: performed the operation. AB, PW, YT, and AD: involved in all congenital cardiac case discussions prior to surgery during the congenital heart team meeting. AS, PW, and YT: drafted the initial version of the manuscript. AD: involved in delivering imaging. All authors: reviewed extensively the draft version of the manuscript after which critical revision has been made. All authors: involved in delivering the key clinical message and have contributed intellectually to the case report.

## ETHICAL APPROVAL

All procedures performed in studies involving human participants were in accordance with the ethical standards of the institutional and/or national research committee and with the 1964 Helsinki declaration and its later amendments or comparable ethical standards.

## INFORMED CONSENT

Informed consent was obtained from all individual participants included in the study.
